# The economic burden of idiopathic pulmonary fibrosis in Australia: a cost of illness study

**DOI:** 10.1007/s10198-022-01538-7

**Published:** 2022-10-27

**Authors:** Ingrid A. Cox, Barbara de Graaff, Hasnat Ahmed, Julie Campbell, Petr Otahal, Tamera J. Corte, Yuben Moodley, Nicole Goh, Peter Hopkins, Sacha Macansh, E. Haydn Walters, Andrew J. Palmer

**Affiliations:** 1 Menzies Institute for Medical Research, University of Tasmania, 17 Liverpool Street, Hobart, TAS Australia; 2 NHMRC Centre of Research Excellence for Pulmonary Fibrosis, Camperdown, Australia; 3 Central Clinical School, The University of Sydney, Camperdown, Australia; 4 Department of Respiratory and Sleep Medicine, Royal Prince Alfred Hospital, Camperdown, Australia; 5 Faculty of Health and Medical Sciences, The University of Western Australia, Perth, Australia; 6 Institute of Respiratory Health, The University of Western Australia, Perth, Australia; 7 Department of Respiratory Medicine, Fiona Stanley Hospital, Murdoch, Australia; 8 Department of Respiratory Medicine and Sleep, Alfred Hospital, Melbourne, Australia; 9 Department of Respiratory and Sleep Medicine, Austin Hospital, Melbourne, Australia; 10 Queensland Centre for Pulmonary Transplantation and Vascular Disease, The Prince Charles Hospital, Chermside, Australia; 11 Faculty of Medicine, University of Queensland, Queensland, Australia; 12 Australian Idiopathic Pulmonary Fibrosis Registry, Lung Foundation of Australia, New South Wales, Australia

**Keywords:** Cost of illness, Idiopathic pulmonary fibrosis, Heath resource utilization, Economic burden

## Abstract

**Purpose:**

Idiopathic pulmonary fibrosis (IPF) is a type of interstitial lung disease found mostly in elderly persons, characterized by a high symptom burden and frequent encounters with health services. This study aimed to quantify the economic burden of IPF in Australia with a focus on resource utilization and associated direct costs.

**Methods:**

Participants were recruited from the Australian IPF Registry (AIPFR) between August 2018 and December 2019. Data on resource utilization and costs were collected via cost diaries and linked administrative data. Clinical data were collected from the AIPFR. A “bottom up” costing methodology was utilized, and the costing was performed from a partial societal perspective focusing primarily on direct medical and non-medical costs. Costs were standardized to 2021 Australian dollars ($).

**Results:**

The average annual total direct costs per person with IPF was $31,655 (95% confidence interval (95% CI): $27,723–$35,757). Extrapolating costs based on prevalence estimates, the total annual costs in Australia are projected to be $299 million (95% CI: $262 million–$338 million). Costs were mainly driven by antifibrotic medication, hospital admissions and medications for comorbidities. Disease severity, comorbidities and antifibrotic medication all had varying impacts on resource utilization and costs.

**Conclusion:**

This cost-of-illness study provides the first comprehensive assessment of IPF-related direct costs in Australia, identifies the key cost drivers and provides a framework for future health economic analyses. Additionally, it provided insight into the major cost drivers which include antifibrotic medication, hospital admissions and medications related to comorbidities. Our findings emphasize the importance of the appropriate management of comorbidities in the care of people with IPF as this was one of the main reasons for hospitalizations.

**Supplementary Information:**

The online version contains supplementary material available at 10.1007/s10198-022-01538-7.

## Introduction

Idiopathic pulmonary fibrosis (IPF) is a form of interstitial lung disease (ILD) characterized by progressive fibrosis of the lung parenchyma which results in a progressive decline in lung function, respiratory failure and eventual death [1]. It more commonly occurs in elderly persons above the age of 60 years, most frequently in males. Median survival time is typically 3–5 years from diagnosis [1, 2]. IPF is usually associated with a high symptom burden, typified by progressively worsening cough and shortness of breath, which have a significant impact on health-related quality of life (HRQoL) [1]. Considering that IPF often affects elderly persons, multiple comorbidities are commonly present, and it is now understood that these some of these comorbidities have an important impact on clinical outcomes [3]. Given the aforementioned, persons with IPF generally have frequent encounters with health services which can place an immense burden on health resources [4].

Disease specific therapeutic options for IPF are limited. Only two antifibrotic medications (pirfenidone and nintedanib) have been approved for persons with mild to moderate IPF [1]. Antifibrotic medication is quite costly with costs varying across countries from $2000 to $14,000 per person per month [4, 5]. These were approved for subsidization 2017 in Australia and incurred costs of approximately $139 million over a 3-year period (2017–2020) [6]. In addition to costly medications, people with IPF may require long-term oxygen, palliative care or lung transplants, all of which are costly [4, 7].

Internationally, there is a limited body of evidence on costs and resource utilization associated with IPF and almost no data from after the approval of antifibrotic treatment [4]. This is also the case in Australia [6]. Quantifying the economic burden of diseases is essential for health-care decision-making, to maximize efficient use of limited resources and to ensure access to essential treatments and services. For this reason, this study sought to characterize the economic burden of IPF in Australia, by assessing the direct costs and associated health care resource utilization over a 12-month period in a cohort of people living with IPF.

## Methods

### Study perspective

This retrospective cost of illness study was part of a broader study under the National Health and Medical Research Council (NHMRC), Centre for Research Excellence for Pulmonary Fibrosis (CRE-PF) [8]. Costs were analyzed for the 12-month period prior to recruitment into the study. A “bottom up” costing methodology was utilized, and the costing was performed from a partial societal perspective (Australian Government and patient) focusing primarily on direct costs, which are costs incurred by the health system, society, family or individual patient [9]. Indirect costs, which include productivity losses due to morbidity and mortality, borne by the individual, family, society, or the employer, were not included in this analysis as most participants were of retirement age [9].

### Study participants

Participants for this study were recruited between August 2018 and December 2019 from the Australian IPF Registry (AIPFR), a national multi-centre, prospective registry of IPF patients facilitated by the Lung Foundation of Australia (LFA) [10, 11]. Details on the recruitment methodology for the AIFPR have been previously described [10]. Participation was voluntary through informed consent, and withdrawal was possible at any time without reason. More details are provided in supplement S1.

### Data sources

Cost diary data were collected via a predesigned paper-based survey instrument, which also collected socio-demographic information, information on treatment and on comorbidities. Clinical information was retrieved from the AIPFR database and included pulmonary function tests (PFT) to ascertain disease severity levels, and body mass index (BMI). Administrative data for health resource utilization and associated costs were obtained via data linkage from the Commonwealth Department of Health, the Centre for Health Record Linkage (CHeReL), the Tasmanian Data Linkage Unit (TDLU) and the Centre for Victorian Data Linkage. Medicare Benefits Schedule (MBS) and Pharmaceutical Benefits Scheme (PBS) were obtained for all participants who consented, while hospital use data were only collected for participants from Victoria (VIC), Tasmania (TAS), New South Wales (NSW), and the Australian Capital Territory (ACT), due to pragmatic considerations regarding multiple ethics applications. Further details on data elements collected can be found in the supplement (Table S1).

### Assessment of disease severity

A number of methods are used to assess disease severity of IPF; however, there is no consensus on a single staging system [12, 13]. Physiologic measurements using pulmonary function test (PFT) parameters such as the forced vital capacity as a percent predicted (FVC%) or diffusion capacity for carbon monoxide (DLco) have been commonly used, as well as composite measures of several physiologic values or demographic features [12, 14–17]. For our study we used the following three methods, the FVC%, and two composite measures, the Composite Physiological Index (CPI) [15, 18] and the Gender, Age, Physiology (GAP) assessment [14].

Thresholds for the FVC% were based on previous studies [14–17] with FVC% > 75% considered as mild disease, FVC% between 50 and 75% as moderate disease, and FVC% < 50% as severe disease. For the CPI we used a predetermined formula [15, 18] based on the FVC%, DLco% and the forced expiratory volume as a percent predicted (FEV%). A CPI ≤ 40 is considered as mild disease and CPI > 40 as moderate to severe disease [15]. GAP staging [14] uses the four parameters gender, age, FVC% and the diffusing capacity of the lungs for carbon monoxide percent predicted (DLco%). A GAP index ranging from 0 to 8 is generated from the summation of the scores from each category. The index then allows for classification into the following stages: Stage I (GAP: 0–3), Stage II (GAP: 4–5) and Stage III (GAP: 6–8), with Stage I being the least severe.

In cases where lung function was not available for participants, we categorized participants as “not classified” but still included them in the analysis.

### IPF medications

For the purpose of this analysis, we classified medications used in the treatment of IPF based on international guidelines [19]. Pirfenidone and nintedanib were categorized as “antifibrotics”, anti-reflux medications were classified as “Other IPF medications (with limited evidence)” and prednisolone, n-acetylcysteine, warfarin and azathioprine as, “medications not recommended for IPF treatment” [19].

### Cost analysis

Costs are analyzed retrospectively for the 12-month period prior to participants’ recruitment (from August 2018 to December 2019). All costs were standardized to Australian dollars ($) 2021, using the Australian Consumer Price Index from the Australian Bureau of Statistics (ABS) [20]. Table 1 provides a summary of the cost categories considered in this analysis.

**Table 1:** Cost categories considered in the analysis

Cost category	Inclusions	Government	Patient
Direct medical costs			
Medication	Antifibrotic medications	x	x
	Other respiratory medications	x	x
	Other prescription medication	x	x
	Non-prescription medication		x
Healthcare professionals	General practitioners	x	x
	Specialists	x	x
	Nursing services	x	x
	Allied professionals	x	x
Medical tests and procedures	Diagnostic tests	x	x
	IPF diagnostics and procedures	x	x
	Non-IPF diagnostics and procedures	x	x
Hospital attendances^a^	Emergency department	x	x
	Admissions	x	x
Special equipment	Mobility aids	x	x
	Oxygen therapy/respiratory	x	x
	Living aids	x	x
Direct non-medical costs	Community services	x	x
	Transportation		x

^a^Patient costs in the case of hospital attendances include out of pocket costs and insurance payment

#### Outcome variables

This study focused primarily on the following four outcome variables: annualized total direct costs, total direct medical costs, total direct non-medical costs and service utilization for major cost drivers. Costs were further stratified by disease severity, participant characteristics and to illustrate the government costs and out of pocket costs (OOP).

#### Medication data

Prescription medication costs and quantity were extracted from the PBS data and were classified based on the Australian Medicines Handbook [21]. For the purposes of this analysis, we categorized prescription medications into the following:(1) antifibrotics; (2) other respiratory medications; and (3) medications for comorbidities. Annual prescription costs were summarized for each participant for the three categories. For this category costs were considered from the government and patient perspective, i.e., OOP costs.

Non-prescription medication costs and quantity were extracted from the cost diaries, and these were reported for a period of 4 weeks. Where costs were not recorded for medications, we estimated an average cost, using costs from the four largest pharmacies in Australia [22]. Monthly costs were extrapolated to annual costs with the assumption that the same utilization patterns were maintained. For this category, only OOP costs were considered.

#### Ambulatory care data

Ambulatory care costs and visits were extracted from MBS data. All services that were not flagged as inpatient services were included in this category and included healthcare professionals and medical and diagnostic tests (Table 1). For this category, costs were considered from the government perspective and patient perspective.

#### Hospital data

Admitted patient costs and visits were derived from the Australian Refined Diagnosis Related Groups (AR-DRGs) classification system for public hospital funding published by Independent Hospital Pricing Authority (IHPA [23]. Private hospital admission costs were estimated using adjustments recommended by the IHPA [23].

Emergency department attendances were costed based on Urgency Related Groups (URGs) [23].

Sub-acute admissions (rehabilitation, palliative, and geriatric evaluation/management care) were costed based on the Australian National Subacute and Non-acute Patient Classification (AN-SNAP) [23].

#### Medical equipment

We estimated costs for medical equipment based on participants’ responses in the cost diary. For consumables/accessories where the time frame for use was not provided, an assumed the life span of 1 month was used [24–26]. Where no participant costs were incurred, we assumed that the equipment was covered by a government agency and categorized it as a government cost.

#### Community services

Community service attendances and costs were based on participants’ responses in the cost diary. Where costs were not provided by participants, we assumed that services were supplied based on hourly rates, in accordance with the National Summary of Home Care Prices and applicable government subsidies [27]. OOP costs were assumed to be the hourly costs less the government and other subsidies including Department of Veterans' Affairs (DVA) [27]. Monthly costs were extrapolated to annual costs.

#### Transportation

Transportation use and costs were based on responses from the cost diary on distance travelled over a 4-week period that was IPF related. Fuel costs per kilometer were based on average fuel consumption patterns for a passenger vehicle and the average national fuel price for 2019 [28, 29]. Other costs included in this category were travel costs to other states for services related to IPF, taxi charges, public transportation, and parking costs. Monthly costs were then extrapolated to annual costs for each participant. For this category, only OOP costs were considered.

#### Statistical analysis

Statistical analyses were conducted using R Software [30]. Characteristics of participants are presented descriptively as means and standard deviations (SD) for continuous variables or counts and proportions for categorical variables. Two sample *t* test or Chi-squared tests were used where appropriate to compare (i) participants in the study and persons in the AIPFR who did not consent to the study and (ii) participants with PFTs and participants with incomplete or missing PFTs. A *p* value less than 0.05 was used as a test for statistical significance.

All costs were summarized as mean costs per person and 95% confidence intervals (95% CI). Summary costs by participants’ characteristics were summarized as mean costs per person (95% CI) and median costs and the interquartile range (IQR). Resource utilization was summarized as the mean (SD) per person and where appropriate as the median per person and IQR.

To estimate total annual population costs, we used a prevalence-based approach [9]. Population estimates were obtained from the ABS [31] and prevalence estimates for IPF were obtained from Cox et al. [32].

Service utilization was summarized as counts and proportions for the 12-month period and means and SDs or 95% CIs where applicable.

Generalized linear models (GLM) with gamma distribution and a log link function were used to model the effect of disease severity and other key sociodemographic and clinical variables on total direct costs [33, 34]. Additionally, we evaluated the effect of these variables on health service utilization, focusing on the major cost drivers, using negative binomial hurdle models [34, 35]. For regression analyses we used the continuous variables for disease severity and BMI. For FVC% and CPI we evaluated the outcome for every 10-unit increase of FVC% and the CPI index and for GAP and BMI every 1-unit increase. We first evaluated univariable models for each variable and then multivariable models based on each disease severity classification.

## Results

### Participant characteristics

Table 2 provides a summary of participant characteristics. From the AIPFR, the following 288 people were invited to participate: 162 participants consented corresponding to a 56% response rate. Persons who did not participate in the study (*n* = 126) had more comorbidities and were older than participants (Supplement Table S2). Participants with lung function tests (*n* = 112) were more likely to be on antifibrotic medications (Table 2).

**Table 2:** Participant characteristics based on lung function tests

	All Participants	PFT	Incomplete/No PFT	*p*
	(*n* = 162)	(*n* = 112)	(*n* = 50)	
Age				0.8
Mean (SD)	73.8 (7.6)	73.7 (7.4)	74.1 (8.2)	
Median [IQR]	74.0 [69–78]	74.0 [69–78]	74.0 [69–78]	
Age group, *n* (%)				0.9
< 65	19 (11.7)	13 (11.6)	6 (12.0)	
65–75	82 (50.6)	56 (50.0)	26 (52.0)	
75–85	48 (29.6)	35 (31.3)	13 (26.0)	
> 85	13 (8.0)	8 (7.1)	5 (10.0)	
Gender, *n* (%)				0.7
Male	99 (61.1)	70 (62.5)	29 (58.0)	
Female	63 (38.9)	42 (37.5)	21 (42.0)	
Race, *n* (%)				0.4
Caucasian	145 (89.5)	100 (89.3)	45 (90.0)	
Other	9 (5.6)	8 (7.1)	1 (2.0)	
Missing	8 (4.9)	4 (3.6)	4 (8.0)	
Marital Status, *n* (%)				**0.04**
Married/De facto/Partner	115 (71.0)	85 (75.9)	30 (60.0)	
Divorced/Widowed/Separated/Single	45 (27.8)	25 (22.3)	20 (40.0)	
Missing	2 (1.2)	2 (1.8)	0 (0.0)	
State of usual residence, *n* (%)				** < 0.001**
NSW	66 (40.7)	57 (50.9)	9 (18.0)	
VIC	31 (19.1)	21 (18.8)	10 (20.0)	
QLD	14 (8.6)	5 (4.5)	9 (18.0)	
SA	25 (15.4)	22 (19.6)	3 (6.0)	
TAS	16 (9.9)	5 (4.5)	11 (22.0)	
WA	6 (3.7)	0 (0.0)	6 (12.0)	
ACT	2 (1.2)	2 (1.8)	0 (0.0)	
NT	2 (1.2)	0 (0.0)	2 (4.0)	
Remoteness area, *n* (%)				0.1
Major city	99 (61.1)	75 (67.0)	24 (48.0)	
Inner regional	43 (26.5)	28 (25.0)	15 (30.0)	
Outer regional/ Remote	16 (9.9)	8 (7.1)	8 (16.0)	
Missing	4 (2.5)	1 (0.9)	3 (6.0)	
Employment, *n* (%)				0.9
Full time/Part time/Unpaid work	19 (11.7)	14 (12.5)	5 (10.0)	
Retired	135 (83.3)	92 (82.1)	43 (86.0)	
Unemployed	7 (4.3)	5 (4.5)	2 (4.0)	
Missing	1 (0.6)	1 (0.9)	0 (0.0)	
Income ($), *n* (%)				0.2
< 400/week	56 (34.6)	39 (34.8)	17 (34.0)	
400–799/week	50 (30.9)	33 (29.5)	17 (34.0)	
800–1249/week	15 (9.3)	8 (7.1)	7 (14.0)	
> 1250/week	12 (7.4)	11 (9.8)	1 (2.0)	
Missing	29 (17.9)	21 (18.8)	8 (16.0)	
Concession status, *n* (%)				
DVA	7 (4.3)	4 (3.6)	3 (6.0)	0.8
Pensioner	107 (66.0)	71 (63.4)	36 (72.0)	0.4
Seniors	39 (24.1)	20 (17.9)	19 (38.0)	**0.01**
Other	24 (14.8)	14 (12.5)	10 (20.0)	0.3
No card	30 (18.5)	25 (22.3)	5 (10.0)	0.1
Comorbidities, *n* (%)				1.0
No	33 (20.4)	23 (20.5)	10 (20.0)	
Yes	129 (79.6)	89 (79.5)	40 (80.0)	
BMI kg/m^2^ (*n* = 154)				0.1
Mean ± SD	28.1 (4.8)	27.6 (4.3)	29.3 (5.8)	
Median [IQR]	28 [25–31]	27 [25–30]	28 [25–33]	
Missing	8 (4.9)	0 (0.0)	8 (16.0)	
GAP stage, (*n* = 108)				** < 0.001**
Stage I	46 (28.4)	46 (41.1)	0 (0)	
Stage II	52 (32.1)	52 (46.4)	0 (0)	
Stage III	10 (6.2)	10 (8.9)	0 (0)	
Not classified	54 (33.3)	4 (3.6)	50 (100)	
FVC, (*n* = 112)				** < 0.001**
> 75	82 (50.6)	82 (73.2)	0 (0.0)	
50–75	26 (16.0)	26 (23.2)	0 (0.0)	
< 50	4 (2.5)	4 (3.6)	0 (0.0)	
Not classified	50 (30.9)	0 (0.0)	50 (100.0)	
CPI, (*n* = 108)				** < 0.001**
< 40	38 (23.5)	38 (33.9)	0 (0.0)	
> 40	70 (43.2)	70 (62.5)	0 (0.0)	
Not classified	54 (33.3)	4 (3.6)	50 (100.0)	
Medications, *n* (%)				
Antifibrotics	96 (59.3)	75 (67.0)	21 (42.0)	** < 0.001**
Other medications for used IPF (limited evidence)	81 (50.0)	54 (48.2)	27 (54.0)	0.61
Medications not recommended for IPF treatment	27 (16.7)	12 (10.7)	15 (30.0)	** < 0.001**

*n* number of participants, *SD* Standard deviation, *$* Australian dollars, *FVC* forced vital capacity percent predicted, *GAP* Gender, Age, Physiology; *CPI* Composite Physiological Index. Not classified (includes participants with missing or incomplete PFTs), *BMI* Body Mass Index, *IQR* interquartile range, Antifibrotics medications include pirfenidone and nintedanib. Medications not recommended for IPF treatment include prednisolone, n-acetylcysteine, warfarin and azathioprine. Other medications used for IPF (limited evidence) includes anti-reflux drugs. *NSW* New South Wales, *VIC* Victoria, *SA* South Australia, *QLD* Queensland, *TAS* Tasmania, *WA* Western Australia, *ACT* Australian Capital Territory, *NT* Northern Territory

*p* value for appropriate test (*t* test or Chi-squared test). Bolded results represent statistically significant results (*p* < 0.05)

Eighty-three percent of participants were retired and 80% were aged 65–85 years. The mean age for participants was 73.8 (7.6) years. Most participants were males (61%), Caucasian (90%), resident of major cities (61%) and were based in New South Wales (41%) which is Australia’s most populous state [31]. Approximately 80% had a comorbidity and 60% were on antifibrotic treatment. Sixty percent of participants had mild-moderate disease based on GAP staging and 67% based on FVC% classification. Based on CPI classification, 43% of participants had moderate to severe disease.

### Costs

**Fig. 1:**
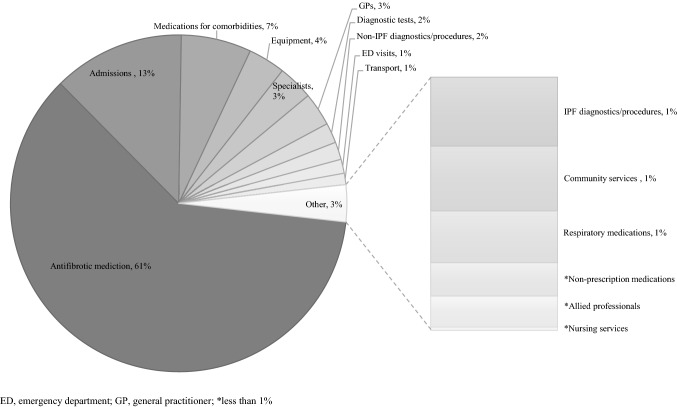
Proportion of total direct costs by cost category. *ED* emergency department, *GP* general practitioner, *less than 1%

Table 3 provides a summary of costs for the 12-month study period. The mean total direct costs per person with IPF were $31,655 (95% CI $27,723–$35,757). The main cost drivers were antifibrotic medication (61% of total direct costs), hospital admissions (13%) and medications for comorbidities (7%) as illustrated in Fig. 1. The mean costs per person for antifibrotic medication, hospital admissions and medications for comorbidities were $19,340 (95% CI $16,238–$22,809), $4026 (95% CI $2590–$6483) and $2146 (95% CI $1602–$3214), respectively. Other high-cost categories included equipment (4%, $1134) which was driven mainly by oxygen concentrators, specialist costs (3%, $1081) and general practitioners (3%, $1021). Mean OOP costs per person for the period were $2,687 (95% CI $2148–$3478), representing 8% of the total costs. Mean government costs were $28,968 (95% CI $25,238–$33,084) representing 92% of the total mean costs per person. When we extrapolated our cost estimates to the population, prevalence-based total annual direct costs for IPF in Australia were $299 million (95% CI $262 million–$338 million), with government costs and OOP costs being $274 million (95% CI $239 million–$313 million) and $25.4 million ($20.3 million–$32.9 million), respectively.

**Table 3:** Resource utilization, government costs and out of pocket costs by cost category for a 12-month period for persons with IPF in Australia

	Resource utilisation			Costs					
	Total (*n*)	Mean per person (95% CI)	Range per person	Mean government costs per person (95% CI) ($)	Mean OOP costs per person (95% CI) ($)	Overall mean costs per person (95% CI) ($)	Total government burden (95% CI) ($ millions)	Total OOP burden (95% CI) ($ millions))	Total overall burden (95% CI) ($ millions)
Medical services									
Medication (prescriptions)									
Antifibrotic	1010	6 (5–7)	1–22	19,255 (16,158–22,715)	86 (65–116)	19,340 (16,238–22,809)	182 (153–215)	0.8 (0.6–1.0)	183 (154–216)
Other respiratory	548	3 (2–5)	1–51	199 (114–472)	32 (21–50)	232 (144–516)	1.8 (1.1–4.5)	0.3 (0.2–0.5)	2.2 (1.4–4.9)
Medications for comorbidities	7763	48 (42–54)	2–172	1,755 (1,235–2,760)	391 (331–472)	2,146 (1,602–3,214)	16.6 (11.7–26.1)	3.7 (3.1–4.5)	20.3 (15.2–30.4)
Non-prescription	138	1 (0–1)	1–7	–	148 (101–236)	148 (101–236)	–	1.4 (1.0–2.2)	1.4 (1.0–2.2)
Healthcare professionals (visits)									
General practitioners	2704	17 (15–19)	3–63	952 (845–1,072)	65 (52–91)	1,021 (917–1,140)	9.0 (8.0–10.1)	0.6 (0.5–0.9)	9.7 (8.7–10.8)
Specialists	1397	9 (7–13)	1–176	820 (576–1,873)	261 (208–353)	1,081 (798–2,079)	7.7 (5.4–17.7)	2.5 (2.0–3.3)	10.2(7.5–19.7)
Nursing services	186	1 (1–2)	1–11	15 (11–19)	–	15 (11–19)	0.1 (0.1–0.2)	–	0.1 (0.1–0.2)
Allied professionals	316	2 (1–3)	1–19	94 (71–128)	17 (9–35)	138 (100–200)	0.8 (0.6–1.2)	0.2 (0.1–0.3)	1.3 (0.9–1.9)
Medical tests and procedures (tests/procedures)									
Diagnostic tests	4812	30 (26–36)	1–302	543 (466–707)	71 (44–118)	614 (517–805)	5.1 (4.4–6.7)	0.7(0.4–1.1)	5.8(4.9–7.6)
IPF diagnostics and procedures	394	2 (2–3)	1–13	290 (252–334)	17 (10–28)	306 (267–352)	2.7 (2.4–3.2)	0.2 (0.1–0.3)	2.9 (2.5–3.3)
Non-IPF diagnostics and procedures	721	4 (4–5)	1–30	482 (392–627)	54 (36–91)	536 (436–708)	4.6 (3.7–5.9)	0.5(0.3–0.9)	5.1(4.1–6.7)
Hospital (visits/admissions)									
Emergency department	75	1 (0–1)	1–8	429 (295–642)	–	429 (295–642)	4.1 (2.8–6.1)	–	4.1 (2.8–6.1)
Admissions	106	1 (0–1)	1–10	3,491 (2,199–5,871)	535 (298–1,073)	4,026 (2,590–6,483)	33.0 (20.8–55.5)	5.1 (2.8–10.2)	38.1 (24.5–61.3)
Equipment	80	1 (0–1)	1–16	484 (197–1,258)	649 (364–1,138)	1,134 (676–2,025)	4.6 (1.9–11.9)	6.1 (3.4–10.8)	10.7(6.4–19.2)
Total				28,836 (25,144–32,913)	2,182 (1,708–2,869)	31,018 (27,260–35,283)	273 (238–311)	20.6 (16.2–27.1)	293 (258–334)
Non-medical services									
Community services (hours)	76	1 (0–1)	1–16	132 (79–239)	157 (93–290)	289 (173–529)	1.2 (0.7–26.1)	1.5(0.9–2.7)	2.7(1.6–5.0)
Transportation (km)	10,275	64 (41–104)	5–1,420	–	347 (197–786)	347 (197–786)	–	3.3 (1.9–7.4)	3.3 (1.9–7.4)
Total				132 (79–239)	505 (325–951)	637 (420–1,101)	1.2 (0.7–26.1)	4.8 (3.1–9.0)	6.0 (4.0–10.4)
Overall total costs				28,968 (25.238–33,084)	2,687 (2,148–3,478)	31,655 (27,723–35,757)	274 (239–313)	25.4 (20.3–32.9)	299 (262–338)

*n* number, *SD* Standard deviation, *IQR* interquartile range, *CI* confidence intervals, *OOP* out of pocket, *$* Australian dollars, *km* kilometers

A closer evaluation of costs based on participant characteristics (Table 4) demonstrated higher mean costs were incurred by persons who were 75–85 years old, persons in outer regional or remote areas and persons with comorbidities had higher costs than those without. In general, there was an increase in mean total direct costs per person with increasing disease severity when evaluated using GAP staging and the CPI index, but this pattern was not seen when disease severity was evaluated using the FVC%. For GAP staging, costs were $28,248 ($21,759–$34,737), $35,563 ($28,043–$43,084) and $39,198 ($19,308–$59,088) for stage 1, 2 and 3, respectively. For the CPI classification, costs were $25,335 ($17,823–$32,847) and $36,828 ($30,750–$42,906) for mild disease and moderate to severe disease, respectively. For the FVC% classification costs were highest for moderate disease, $34,596 ($23,097–$46,095) with costs for mild and severe disease being $31,095 ($25,730–$36,460) and $28,710 ($0–$63,084), respectively. Persons on antifibrotic medication had the highest costs when compared to persons not on antifibrotic medication or on other regimens related to IPF treatment.

**Table 4:** Total costs by participant characteristics for a 12-month period for persons with IPF in Australia

	*n*	Mean costs per person (95% CI) ($)	Median costs per person (IQR) ($)	Range (min–max) ($)
Age group	162			
< 65	19	25,865 (16,737–34,993)	19,395 (14,616–35,297)	5135–75,096
65–75	82	29,894 (24,631–35,158)	29,462 (9,366–46,291)	0–110,215
75–85	48	39,380 (30,452–48,309)	40,066 (9652–57,619)	90–127,833
> 85	13	22,696 (6429–38,962)	9,323 (2734–37,263)	0–82,254
Gender				
Male	99	34,342 (28,784–39,901)	31,023 (8409–52,049)	0–127,833
Female	33	27,431 (21,629–33,234)	23,749 (9417–40,696)	0–110,215
Remoteness area				
Major city	99	31,499 (26,551–36,446)	29,172 (9352–48,099)	0–98,483
Inner regional	43	30,589 (23,176–38,001)	28,791 (8780–50,719)	0–85,132
Outer regional/remote	16	42,146 (21,978- 62,314)	37,886 (12,152- 52,505)	114–127,833
Comorbidities				
No	33	31,741 (24,350–16,958)	18,494 (5577–39,537)	114- 78,809
Yes	129	38,263 (33,523–28,783)	29,561 (9382–51,087)	0–127,833
BMI kg/m^2^ (*n* = 154)				
Normal	39	31,137 (23,248–39,026)	27,956 (11,175–31,137)	0–85,132
Underweight	§	§	§	§
Overweight	66	31,358 (24,963–37,752)	29,903 (7752–31,358)	0–98,483
Obese	48	32,473 (24,863–40,083)	29,267 (10,161–32,473)	90–110,215
FVC%, (*n* = 112)				
> 75	82	31,095 (25,730–36,460)	28,937 (8265–31,095)	0–98,483
50–75	26	34,596 (23,097–46,095)	31,570 (11,542–34,596)	0–92,562
< 50	4	28,710 (– 5664 to 63,084)	23,357 (14,585–28,710)	9742–58,382
Not classified	50	31,279 (23,096–39,462)	24,895 (9348–31,279)	0–127,833
GAP stage, (*n* = 108)				
Stage I	46	28,248 (21,759–34,737)	25,124 (9156–28,248)	179–75,096
Stage II	52	35,563 (28,043–43,084)	36,563 (10,157–35,563)	0–98,483
Stage III	10	39,198 (19,308–59,088)	38,790 (17,086–39,198)	1,871–79,000
Not classified	54	29,396 (21,618–37,173)	19,885 (6563–29,396)	0–127,833
CPI, (*n* = 108)				
< 40	38	25,335 (17,823–32,847)	19,620 (6859–25,335)	0–75,096
> 40	70	36,828 (30,750–42,906)	35,491 (16,329–36,828)	0–98,483
Not classified	54	29,396 (21,618–37,173)	19,885 (6563–29,396)	0–127,833
Medications				
Antifibrotics				
No	66	17,237 (12,682–21,791)	9,597 (4247–22,988)	0–85,132
Yes	96	41,567 (36,250–46,883)	40,699 (24,987–55,810)	0–127,833
Other medications for used IPF (limited evidence)				
No	81	28,725 (23,072–34,378)	23,749 (6204–42,976)	0–98,483
Yes	81	34,584 (28,672–40,496)	31,023 (10,792–51,087)	0–127,833
Medications not recommended for IPF treatment				
No	135	31,964 (27,322–36,607)	29,362 (7768–49,426)	0–127,833
Yes	27	30,106 (21,923–38,289)	23,749 (12,993–43,840)	939–82,254

*n* number of participants, *$* Australian dollars, *CI* confidence intervals, *min* minimum, *max* maximum, *FVC* forced vital capacity percent predicted, *GAP* Gender, Age, Physiology; *CPI* Composite Physiological Index. Not classified, includes participants with missing or incomplete PFTs; *BMI* Body Mass Index, *IQR* interquartile range, Antifibrotics medications include pirfenidone and nintedanib. Medications not recommended for IPF treatment include prednisolone, n-acetylcysteine, warfarin and azathioprine. Other medications used for IPF (limited evidence) includes anti-reflux drugs §, *n* < 5, not able to report due to ethics restriction

Disaggregation of the total direct costs by medical and non-medical components indicated that direct medical costs accounted for 98% of the total direct costs. Mean direct medical costs and mean direct non-medical costs per person were $31,018 ($27,260–$35,283) and $637 ($420–$1101), respectively. Seventy-nine percent of direct non-medical costs were OOP costs, with the majority of these attributable to transportation costs (Table 2).

Regression analyses (Table 5) demonstrated consistent results across the univariable and each of the multivariable models (based on the disease severity classification systems) for comorbidities, disease severity and antifibrotic medication variables. In the multivariable models, our results demonstrated that costs were reduced by a factor of 0.92 of the mean costs for each 10-unit increase in FVC%, for CPI a 10-unit increase corresponded to 1.17-fold increase in mean costs, and for GAP a 1-unit increase corresponded to 1.15-fold increase in mean costs. Participants with comorbidities had a 1.49– to 1.53-fold increase in mean costs compared to persons without comorbidities. Persons on antifibrotics had a 2.66–2.80-fold increase in mean costs when compared to persons not taking antifibrotics. For the FVC% multivariable model, we saw a 1.69-fold increase in costs for the age group 65–75 years, but this was not consistent with the GAP or CPI multivariable models. Likewise, for the GAP model we observed a 1.58-fold increase in mean costs in persons on treatment regimens that are not recommended when compared to those who were not on these regimens.

**Table 5:** Regression analysis: factors influencing total direct costs

	Exponentiated univariable coefficient (95% CI)	GAP Exponentiated multivariable coefficient	FVC Exponentiated multivariable coefficient	CPI Exponentiated multivariable coefficient
Age group				
< 65	Reference	Reference	Reference	Reference
65–75	1.16 (0.75–1.72)	1.44 (0.85–2.35)	**1.69 (1.02–2.72)**	1.58 (0.94–2.54)
75–85	1.52 (0.97–2.33)	1.36 (0.80–2.25)	1.55 (0.92–2.55)	1.48 (0.87–2.44)
> 85	0.88 (0.49–1.59)	0.77 (0.40–1.52)	1.32 (0.67–2.65)	0.97 (0.50–1.93)
Gender				
Male	Reference	Reference	Reference	Reference
Female	0.99 (0.62–1.04)	0.90 (0.64–1.27)	0.89 (0.66–1.22)	0.88 (0.65–1.20)
Remoteness area				
Major city	Reference	Reference	Reference	Reference
Inner regional	0.97 (0.73–1.30)	1.12 (0.80–1.59)	1.18 (0.84–1.67)	1.21 (0.86–1.73)
Outer regional/remote	1.34 (0.89–2.08)	0.87 (0.54–1.46)	0.93 (0.57–1.59)	0.87 (0.54–1.47)
Comorbidities				
No	Reference	Reference	Reference	Reference
Yes	**1.38 (1.00–1.87)**	**1.49 (1.03–2.12)**	**1.52 (1.04–2.18)**	**1.53 (1.06–2.16)**
BMI kg/m^2^	1.01 (0.98–1.03)	1.02 (0.99–1.05)	1.01 (0.98–1.04)	1.02 (0.99–1.05)
GAP total (1-unit increase)	**1.15 (1.04–1.28)**	**1.15 (1.01–1.32)**	–	–
FVC% (10-unit increase)	**0.93 (0.88–0.99)**	–	**0.92 (0.85–0.99)**	–
CPI index (10-unit increase)	**1.22 (1.09–1.35)**	–	–	**1.17 (1.05–1.31)**
Medications (reference = no)				
Antifibrotics	**2.41 (1.85–3.13)**	**2.67 (1.93–3.68)**	**2.80 (2.02–3.88)**	**2.66 (1.92–3.67)**
Other medications for used IPF (limited evidence)	0.94 (0.68–1.35)	1.11 (0.84–1.48)	1.04 (0.78–1.38)	1.10 (0.82–1.46)
Medications not recommended for IPF treatment	1.20 (0.93–1.56)	**1.58 (1.05–2.46)**	1.34 (0.91–2.02)	1.48 (0.97–2.32)
Intercept		5388 (2795–10,780)	9906 (5499–18,595)	7187 (3979–13,530)

*CI* confidence intervals, *min* minimum, *max* maximum, *FVC* forced vital capacity percent predicted, *GAP* Gender, Age, Physiology; *CPI* Composite Physiological Index. Not classified, includes participants with missing or incomplete PFTs; *BMI* Body Mass Index, *IQR* interquartile range; Antifibrotics medications include pirfenidone and nintedanib. Medications not recommended for IPF treatment include prednisolone, n-acetylcysteine, warfarin and azathioprine. Other medications used for IPF (limited evidence) includes anti-reflux drugs; Bolded results represent statistically significant results (*p* < 0.05)

Predicted total per person costs for a hypothetical male aged between 65 and 75 years old with no comorbidities ranged between $22,815 ($15,367–$33,871) and $32,552 ($18,377–$56,602) for GAP classification, between $25,124 ($17,996–$35,075) and $30,784 ($13,032–$72,719) for FVC classification and between $21,852 ($14,976–$31,884) and $29,499 ($20,744–$41,950) for the CPI classification. Table S4 provides further details on selected hypothetical groupings.

### Resource utilization

Table 3 provides a summary of resource utilization for this cohort. Medications for comorbidities accounted for the highest rate of prescriptions (82%), followed by antifibrotic medications (11%). GP visits (59%) and specialist visits (30%) were the most frequent encounters with healthcare professionals. Diagnostic tests were the most used resource in the “medical tests and procedures” category, and non-IPF diagnostics and procedures were more frequently used than IPF diagnostics and procedures. A total of 75 emergency department visits and 106 hospital admissions occurred during the 12-month period with an average of 1 admission/visit per person. On average, 1 piece of home use equipment related to IPF care was acquired, 1 h of community service care was used, and 64 km were travelled for services related to IPF, per person in the cohort per year.

As hospital admissions were of high-cost category, we undertook a more detailed analysis of those. Most admissions were for respiratory diseases (35%), followed by circulatory system diseases (13%) and digestive system diseases (8%). This is in keeping with the comorbidity profile of the participants (Supplement Table S3). Sixty-four percent of all admissions were for comorbidities. The total length of stay (LOS) over the 12-month period for respiratory diseases was 120 days with a mean LOS of 3 days, for circulatory diseases the total number of days was 59 with a mean LOS of 4 days and for digestive system diseases the total was 11 with a mean LOS of 1 day. Nervous system admissions were the fifth most frequent reason for admission but contributed a total of 43 days. On average persons with nervous system disorders were admitted for 7 days, with the longest stay being 28 days. While respiratory disease admissions were the most frequent cause of admission, they did not incur the highest total costs or mean costs per admission. Circulatory system diseases admissions incurred the highest mean costs per admission and total costs. Table 6 provides more details.

**Table 6:** Admitted patient presentations, length of stay and costs by major diagnostic categories for 12-month period for persons with IPF in Australia

Major diagnostic categories (MDC)	Number of admissions, *n* (%)	Length of stay (days)			Cost	
		Total	Mean (SD)	Max	Total ($)	Mean (SD) ($)
Respiratory system	37 (35)	120	3 (3)	10	182,719	4938 (2269)
Circulatory system	14 (13)	59	4 (5)	20	201,555	14,397 (17,046)
Digestive system	8 (8)	11	1 (1)	2	20,323	2540 (1446)
Ear, nose, mouth and throat	7 (7)	13	2 (1)	4	11,803	1686 (684)
Eye	7 (7)	7	1 (1)	1	22,451	3207 (1947)
Infectious and parasitic diseases	6 (6)	11	2 (1)	4	25,467	4245 (2394)
Nervous system	6 (6)	43	7 (11)	28	40,653	6776 (7243)
Kidney and urinary tract	4 (4)	5	1 (1)	2	20,443	5111 (3561)
Musculoskeletal system and connective tissue	4 (4)	4	1 (1)	1	13,083	3271 (1868)
Endocrine, nutritional and metabolic diseases and disorders	3 (3)	4	1 (1)	2	5838	1946 (685)
Factors influencing health status and other contacts with health services	3 (3)	3	1 (1)	1	5543	1848 (695)
Hepatobiliary system and pancreas	2 (2)	2	1 (1)	1	6757	3379 (2657)
Skin, subcutaneous tissue & breast	2 (2)	6	3 (1)	3	11,150	5575 (2269)
Blood and blood forming organs and immunological disorders	1 (1)	1	–	1	979	–
Major procedures where the principal diagnosis may be associated with any MDC	1 (1)	25	–	25	34,026	–
Neoplastic disorders (haematological and solid neoplasms)	1 (1)	5	–	5	17,629	–

A closer look at admissions based on participant characteristics (Table 7) demonstrated that the mean number of admissions were higher in persons older than 85 years, males, persons living in outer regional or remote areas and who were obese. Participants with comorbidities had a higher mean number of admissions than those without comorbidities. Eleven percent of participants with comorbidities had 1 admission and 19% had more than 1 admission. Persons who were not on antifibrotic medication had a higher mean number of admissions and similarly persons on regimens not recommended for treatment of IPF had a higher number of admissions. The CPI classification for disease severity demonstrated that persons with moderate/severe disease had a higher mean number of admissions than persons with mild disease. GAP and FVC% disease classification did not demonstrate this pattern.

**Table 7:** Admissions by participant characteristics for a 12-month period for persons with IPF in Australia

	*n*	Mean number admissions per person (95% CI)	Participants with 1 admission, *n* (%)	Participants with more than 1 admission, *n* (%)	Range (min–max)
All	162	0.65 (0.48–0.91)	21 (13)	27 (17)	0–10
Age group					
< 65	19	0.47 (-0.02–0.97)	3 (16)	2 (11)	0–4
65–75	82	0.54 (0.22–0.86)	8 (10)	10 (12)	0–10
75–85	48	0.79 (0.43–1.15)	8 (17)	11 (23)	0–5
> 85	13	1.15 (0.14–2.17)	2 (15)	4 (31)	0–5
Gender					
Male	99	0.68 (0.42–0.94)	11 (11)	18 (18)	0–6
Female	33	0.62 (0.25–0.99)	10 (16)	9 (14)	0–10
Remoteness area					
Major city	99	0.53 (0.32–0.73)	12 (12)	15 (15)	0–5
Inner regional	43	0.84 (0.39–1.29)	7 (16)	9 (21)	0–6
Outer regional/ Remote	16	1.13 (-0.26–2.51)	2 (12)	3 (19)	0–10
Comorbidities					
No	33	0.36 (0.12–0.61)	7 (21)	2 (6)	0–3
Yes	129	0.73 (0.47–0.99)	14 (11)	25 (19)	0–10
BMI kg/m^2^ (*n* = 154)					
Normal	39	0.49 (0.13–0.84)	7 (18)	4 (10)	0–6
Underweight	§	§	§	§	§
Overweight	66	0.65 (0.38–0.92)	9 (14)	13 (20)	0–4
Obese	48	0.75 (0.22–1.28)	5 (10)	7 (15)	0–10
FVC%, (*n* = 112)					
> 75	82	0.49 (0.29–0.69)	14 (17)	10 (12)	0–4
50–75	26	0.58 (0.05–1.10)	2 (8)	4 (15)	0–5
< 50	4	0.25 (-0.55–1.05)	1 (25)	0 (0)	0–1
Not classified	50	1.00 (0.45–1.55)	4 (8)	13 (26)	0–10
GAP stage, (*n* = 108)					
Stage I	46	0.35 (0.15–0.55)	9 (20)	3 (7)	0–3
Stage II	52	0.69 (0.34–1.05)	6 (12)	10 (19)	0–5
Stage III	10	0.30 (-0.18–0.78)	1 (10)	1 (10)	0–2
Not classified	54	0.94 (0.43–1.45)	5 (9)	13 (24)	0–10
CPI, (*n* = 108)					
< 40	38	0.32 (0.10–0.53)	7 (18)	2 (5)	0–3
> 40	70	0.61 (0.34–0.89)	9 (13)	12 (17)	0–5
Not classified	54	0.94 (0.43–1.45)	5 (9)	13 (24)	0–10
Medications					
Antifibrotics					
No	66	0.73 (0.39–1.06)	10 (15)	12 (18)	0–6
Yes	96	0.60 (0.32–0.89)	11 (11)	15 (16)	0–10
Other medications for used IPF (limited evidence)					
No	81	0.43 (0.24–0.63)	11 (14)	10 (12)	0–5
Yes	81	0.88 (0.50–1.25)	10 (12)	17 (21)	0–10
Medications not recommended for IPF treatment					
No	135	0.59 (0.37–0.81)	18 (13)	20 (15)	0–10
Yes	27	0.96 (0.31–1.62)	3 (11)	7 (26)	0–6

*n* number of participants, *%* percentage, *CI* confidence intervals, *min* minimum, *max* maximum, *FVC* forced vital capacity percent predicted, *GAP* Gender, Age, Physiology; *CPI* Composite Physiological Index. Not classified, includes participants with missing or incomplete PFTs; BMI, Body Mass Index; Antifibrotics medications include pirfenidone and nintedanib. Medications not recommended for IPF treatment include prednisolone, n-acetylcysteine, warfarin and azathioprine. Other medications used for IPF (limited evidence) includes anti-reflux drugs §, *n* < 5, not able to report due to ethics restriction

Table 8 provides a summary of the hurdle regression analysis for hospital admissions. When the odds ratios for hospitalization were assessed, there were no statistically significant relationships, meaning that the probability of having an admission versus not having an admission was not different between participant sub-groups. When the length of stay and number of admissions was evaluated in those subjects who had an admission, comorbidities had a statistically significant impact. The mean number of admissions increased by a 4.07– to 6.55-fold for persons with comorbidities when compared to those without. Univariable models for GAP, FVC% and CPI index also demonstrated statistically significant count part models. For the GAP disease classification multivariable model, there was a 1.53-fold increase in the mean number of admissions for each 1-unit increase in GAP index. For the FVC% disease classification multivariable model, there was a 0.83-fold decrease in the mean number of admissions for each 10-unit increase in FVC%. For the CPI disease classification multivariable model, there was a 1.43-fold increase in the mean number admissions for each 10-unit increase in CPI index. This significant relationship only persisted in the GAP disease classification multivariable model.

**Table 8:** Regression analysis: factors influencing hospital admissions

	Univariable Model		Multivariable models					
			GAP		FVC%		CPI	
	Odds ratio (95% CI)	Admission rate ratio (95% CI)	Odds ratio (95% CI)	Admission rate ratio (95% CI)	Odds ratio (95% CI)	Admission rate ratio (95% CI)	Odds ratio (95% CI)	Admission rate ratio (95% CI)
Age group								
< 65	Reference	Reference	Reference	Reference	Reference	Reference	Reference	Reference
65–75	0.79 (0.25–2.48)	1.80 (0.43–7.45)	0.96 (0.24–3.84)	0.65 (0.14–2.96)	0.78 (0.22–2.73)	1.77 (0.57–5.56)	0.85 (0.22–3.25)	1.61 (0.39–6.63)
75–85	1.83 (0.57–5.93)	1.25 (0.30–5.24)	1.62 (0.39–6.65)	0.49 (0.12–1.91)	1.74 (0.47–6.37)	1.08 (0.35–3.34)	1.34 (0.34–5.32)	1.12 (0.30–4.21)
> 85	2.40 (0.54–10.69)	1.87 (0.36–9.64)	2.20 (0.37–13.09)	0.37 (0.06–2.21)	2.76 (0.50–15.13)	1.81 (0.48–6.77)	2.57 (0.44–15.13)	1.17 (0.23–5.95)
Gender								
Male	Reference	Reference	Reference	Reference	Reference	Reference	Reference	Reference
Female	1.04 (0.52–2.08)	0.80 (0.36–1.78)	1.05 (0.40–2.76)	1.06 (0.54–2.09)	1.15 (0.50–2.65)	1.09 (0.57–2.08)	1.31 (0.54–3.17)	0.80 (0.37–1.74)
Remoteness area								
Major city	Reference	Reference	Reference	Reference	Reference	Reference	Reference	Reference
Inner regional	1.58 (0.74–3.38)	1.34 (0.61–2.93)	1.23 (0.50–3.01)	0.89 (0.38–2.09)	1.20 (0.51–2.84)	1.43 (0.67–3.07)	1.39 (0.56–3.44)	1.19 (0.50–2.80)
Outer regional/Remote	1.21 (0.39–3.81)	2.70 (0.94–7.75)	1.19 (0.32–4.50)	1.01 (0.40–2.55)	1.08 (0.29–3.98)	1.81 (0.74–4.41)	1.29 (0.34–4.93)	1.47 (0.56–3.88)
Comorbidities								
No	Reference	Reference	Reference	Reference	Reference	Reference	Reference	Reference
Yes	1.16 (0.49–2.71)	**4.07 (1.08–15.27)**	0.87 (0.32–2.37)	**5.96 (1.28–27.75)**	0.75 (0.29–1.98)	**4.35 (1.30–14.51)**	0.86 (0.31–2.32)	**6.55 (1.38–31.20)**
					–	–		
BMI kg/m^2^	1.00 (0.93–1.08)	1.03 (0.98–1.14)	1.00 (0.92–1.09)	1.05 (0.96–1.14)	1.02 (0.95–1.11)	1.00 (0.94–1.06)	1.00 (0.92–1.09)	1.03 (0.95–1.12)
GAP total	0.95 (0.72–1.27)	**1.53 (1.08–2.15)**	0.95 (0.66–1.37)	**1.76 (1.20–2.58)**	–	–	–	–
FVC% (10-unit increase)	0.94 (0.81–1.10)	**0.83 (0.69–0.99)**	–	–	0.94 (0.78–1.14)	0.90 (0.75–1.07)	–	–
CPI index (10-unit increase)	1.08 (0.83–1.40)	**1.43 (1.03–1.98)**	–	–			1.09 (0.80–1.47)	1.40 (0.98–2.00)
Medications (reference = no)								
Antifibrotics	0.74 (0.38–1.47)	1.04 (0.48–2.26)	0.76 (0.33–1.75)	0.73 (0.34–1.56)	0.76 (0.34–1.70)	0.97 (0.50–1.87)	0.67 (0.29–1.54)	0.87 (0.40–1.89)
Other medications for used IPF (limited evidence)	1.43 (0.72–2.82)	2.36 (1.10–5.10)	1.52 (0.7–3.32)	1.71 (0.88–3.34)	1.42 (0.66–3.06)	1.81 (0.9–3.63)	1.59 (0.72–3.54)	1.38 (0.64–2.99)
Medications not recommended for IPF treatment	1.50 (0.63–3.57)	1.45 (0.59–3.55)	0.99 (0.33–2.98)	**3.23 (1.13–9.18)**	1.04 (0.37–2.92)	1.34 (0.63–2.86)	0.74 (0.23–2.36)	2.05 (0.74–5.63)
Intercept			0.38 (0.06–2.52)	0.08 (0.01–0.47)	0.53 (0.1–2.85)	0.27 (0.04–1.63)	0.26 (0.03–2.20)	0.04 (0–0.40)

*CI* confidence intervals, *min* minimum, *max* maximum, *FVC* forced vital capacity percent predicted, *GAP* Gender, Age, Physiology; *CPI* Composite Physiological Index. Not classified, includes participants with missing or incomplete PFTs, *BMI* Body Mass Index, *IQR* interquartile range; Antifibrotics medications include pirfenidone and nintedanib. Medications not recommended for IPF treatment include prednisolone, n-acetylcysteine, warfarin and azathioprine. Other medications used for IPF (limited evidence) includes anti-reflux drugs; Bolded results represent statistically significant results (*p* < 0.05)

## Discussion

Our study is the first to provide a comprehensive analysis of costs and service utilization for Australians with IPF. Given the importance of nationally relevant economic data for decision making related to health financing and resource allocation in public health, this study has addressed important evidence gaps. Our results demonstrated that the average total direct costs per person for a 12-month period were $31,655, 98% of which were direct medical costs. The total prevalence-based costs of IPF in Australia were $299 million. This cost estimate was mainly driven by antifibrotic medication, medications for comorbidities and hospital admissions. Participants had frequent encounters with GPs and specialists and had numerous diagnostic tests and prescriptions related to comorbidities. Disease severity, comorbidities and antifibrotic medication all had an influence on costs. The main cost driver for direct non-medical costs were transportation costs. Ninety-two percent of costs were incurred by the Australian government with OOP costs accounting for 8% of the total direct costs.

We reported total direct costs per person of $31,655 for a 12-month period. When compared to the average per capita health expenditure for Australia for 2019 ($7927, 2021 dollars) [36], our estimate is approximately four times this average. Additionally, when compared to published per capita health expenditure for the age group 65–74 years, our estimated total direct costs for males and females were 2.2 and 1.6 times more than the average per capita health costs for this age group [37]. A comparison with IPF resource costs per person in other countries showed that expenditure which included antifibrotic medication was higher in the United States (USA), Canada, France, Belgium, Germany, Spain and Greece than in Australia [38–44] (Supplement Table S5). Comparison of the costs reported in these studies to the respective per capita national health expenditure [45] demonstrated that the ratio of costs in other countries was higher than for Australia. The ratios ranged from 5 times the average national health expenditure in Belgium and Canada, to as much as 16-fold higher in the USA [38–44] (Supplement Table S5). Consideration should, however, be given to varying practice guidelines and payer systems when considering these differences. Further comparison with other disease conditions that are common in this age group in Australia [36, 46–49] illustrated that the ratio between the costs per person and national average health expenditure was lower than the ratio for IPF for persons with asthma, chronic obstructive pulmonary disease (COPD), cardiovascular disease, osteoarthritis, or type II diabetes. This ratio was, however, higher for lung cancer than for IPF. These were also some of the comorbidities encountered in this cohort, acting as an additive factor for cost, as we have demonstrated higher costs in persons with comorbidities when compared to those without. We also observed that costs increased with increasing disease severity. With GAP and CPI classifications, persons with severe disease incurred the highest cost. It must, however, be noted that persons in this group would not generally qualify for antifibrotic medication (the most expensive category) under current guidelines unless they were started when the disease was less severe and hence persons with severe disease incur significant costs, which we noted were mostly driven by hospital admissions. In view of the aforementioned, it is evident that IPF poses a substantial cost burden more specifically, when compared to other respiratory conditions and other diseases of this age group.

Total direct costs were primarily driven by direct medical costs, with antifibrotic medication, hospital admissions and medications for comorbidities being the main cost drivers. There are few published studies which have comprehensively analysed the cost of IPF since the introduction of antifibrotic therapy and those which have, have primarly focused on costs associated with hospitalisations and the associated resource use. Despite this, our study results display general concordance with the limited published literature which identifies transplants, IPF specific medication, admissions and treatment of comorbidities as the key cost generating categories in the management of IPF [4]. Our study, however, did not include participants who had undergone transplants. Antifibrotic medications are known to be costly, and costs can vary between countries from $2000 to $14,000 per person per month [4, 5] dependent of pricing arrangements and payer systems. These medications have been proven to decrease IPF and respiratory related admissions by as much of 45% [50]. While this may be true, the burden of hospitalizations may also be driven by other factors such, medication interactions and general decline in the overall health [4]. The general decline in health may be due to multiple comorbidities which are frequent in this age group [4], and in our cohort (80%). In our cohort, 65% of all admissions were for comorbidities. The type and number of comorbidities were also contributing factors to costs. Forty-seven percent of participants had cardiovascular disease and 28% also had diabetes, both of which are associated with an increase in hospitalizations and associated costs [49]. Evaluating the influence of comorbidity types and combinations on costs will be a focus of our future research.

Our results demonstrated that participants had a high number of encounters with GPs and specialists as well as having numerous diagnostic tests and prescriptions related to medications for comorbidities. [51]. Polypharmacy is a frequent characteristic of multimorbidity, and this was evident as prescription medications for comorbidities were the most frequently supplied medications, in line with our participant comorbidity profile. Studies have shown that IPF patients have a higher need for health professionals and tests than patients without IPF, due to the high number of IPF clinical issues and comorbidities, adverse events related to antifibrotic medications and polypharmacy and the need for monitoring antifibrotic treatment [4, 52]. The introduction of the multidisciplinary team in the management of IPF also increases encounters with the varying health professionals that constitute the team [4]. Transportation was also a frequently used resource in the non-medical category. This is probably an indication of access to services and more specifically access to respiratory physicians. The number of specialists and waiting time to see a specialist varies by jurisdiction and waiting times can be longer in regional and remote areas when compared to major cities [53]. This may result in persons accessing specialist services further than their immediate surroundings. This is especially true for persons who are eligible for transplants as these services are only available in a few jurisdictions. This has been highlighted in a recent qualitative study in Australia [7].

The Australian health system was established on the premise of universal access, and this was more than evident in this analysis, as 92% of costs were covered by government sources. Not much literature is available on OOP costs for IPF [39], but a comparison with the average OOP cost in 2019 in Australia demonstrated that the average OOP for IPF was 1.6-times higher and represents 3.1% of the average income in Australia [36]. While this may seem small, this was based on the average income and not the average retiree’s income and this may still pose a significant strain on households. Despite universal access, health OOP expenses can still have an influence on patients’ ability to maintain other expenses in the household especially when retired. Studies have shown that up to 25–30% of seniors in Australia experience moderate to severe financial burden as a result of their healthcare expenses and as much as 78% reported hardship in a patient population of persons with COPD in NSW [54, 55]. It should be noted that this study did not include costs for admissions into aged care facilities, as there were no persons admitted at the time of collecting the survey. This may be as a result of persons with more severe disease being less likely to respond to the survey.

Our descriptive statistical analysis demonstrated that disease severity, comorbidities and antifibrotic use had an influence on costs and this was confirmed by the regression analysis. Comorbidities, antifibrotic treatment and increasing disease severity all increased costs. When we looked at resource use and more specifically at hospitalizations which accounted for the highest costs, we saw that comorbidities had the greatest effect. While there was no statistically significant difference between the probability of being admitted whether the participant had a comorbidity or not, when admitted the mean number of admissions was higher in persons with comorbidities than those without. The fact that the odds for admission were not statistically different may be due to improved models of care for persons with chronic complex conditions in Australia including care plans for selfcare and management [56], use of outpatient and GP services. Osteoarthritis was also one of the most frequent comorbidities and this is mostly self-managed or managed in primary care. More investigation into the complex relationships between comorbidity and IPF costs and resource utilization is needed and is the focus of our future work.

This study generated cost estimates for a cohort of Australians living with IPF. There were, however, some limitations. First, our sample size was small but given that IPF is a rare and severe disease, this was expected. Based on the estimated number of persons with IPF in Australia, our sample size represents an 8% margin of error with 95% confidence level [32]. This was also evident with the large confidence intervals on some of the cost estimates especially in subgroups with few participants. Following on from this, the representiveness of the sample may affect the accuracy of estimates. We acknowledge that certain subgroups especially older and more severely affected individuals may disproportionately represented affecting the estimates. While we acknowledge this limitation and the margin of error, we are confident that given the uniqueness of the AIPFR registry that we are including a good representation of the Australian population. The AIPFR is based on national collaboration between respiratory physicians both within major public hospitals and the private service [15]. Notwithstanding this, we are cognizant that a triangulation method to provide concrete evidence is necessary which we have included as part of our future research. Third, while most of the costing was based on administrative data, we also used a retrospective cost diary to obtain some of our category estimates, namely transportation, non-prescription medications and community services which may be subject to recall bias. Another limitation to the analysis was the aggregated format of the administrative data which did not allow a microanalysis of factors such as ICU costs. In addition, our prevalence values used in this analysis were based on estimates derived from IPF diagnosis based on International Classification of Diseases (ICD), and this may be slightly higher than actual diagnosed cases based on international guidelines [32]. Finally, we did not include indirect costs in our analysis as most of our cohort was retired, deaths as we conducted a retrospective analysis 12 months prior to the completion of the survey or seasonal variability Including this would have provided a more comprehensive estimate, and we will look further into assessing this appropriately in our cohort in future studies. Despite these limitations, our estimates will be a useful input for health economic evaluations, especially given the estimation of costs for disease severity.

## Conclusion

This cost-of-illness study is the first study to provide comprehensive direct costs related to IPF management in Australia and provides a framework for future health economics studies/evaluation. It also addresses current evidence gaps which are important for reimbursement and policy decisions related to IPF management. Additionally, it provided insight into the major cost drivers which include antifibrotic medication, hospital admissions and medications related to comorbidities. Furthermore, it emphasizes the importance of early diagnosis, early initiation of antifibrotic therapy and the appropriate management of comorbidities in the care of people with IPF as they all have a significant impact on the well-being of persons living with the disease.

## Supplementary Information

Below is the link to the electronic supplementary material.

Supplementary file1 (DOCX 37 KB)

## Data Availability

The datasets generated and code used for the analysis are available from the corresponding author upon reasonable request.
