# Supplementary material for: The economic burden of idiopathic pulmonary fibrosis in Australia: a cost of illness study
Source: Eur J Health Econ. 2022 Oct 27;24(7):1121–39. doi: 10.1007/s10198-022-01538-7 (PMC10406709; doi:10.1007/s10198-022-01538-7)
Supplement: Supplementary file 1 — Supplementary file1 (DOCX 37 KB) [file 10198_2022_1538_MOESM1_ESM.docx]

**Supplement**

**Supplement S1: Methods**

Table S1 Details of data sources used in the analysis

| **Source** | **Elements** | **Time period** | **Participants** |
| --- | --- | --- | --- |
| Cost diary | Prescription medication | 1 month | All |
|  | Non-prescription medication | 1 month | All |
|  | Community services | 1 month | All |
|  | Transport | 1 month | All |
|  | Equipment | 1 year | All |
| Commonwealth Department of Health | MBS | January 2015-June 2019 | All |
|  | PBS | January 2015-June 2019 | VIC, TAS, NSW, ACT |
| Tasmanian Data Linkage Unit | [Admitted Patient Data Collection](https://www.cherel.org.au/data-dictionaries) | January 2015-June 2019 | VIC, TAS, NSW, ACT |
|  | [Emergency Department Data Collection](https://www.cherel.org.au/data-dictionaries) | January 2015-June 2019 | VIC, TAS, NSW, ACT |
| Centre for Victorian Data Linkage | [Admitted Patient Data Collection](https://www.cherel.org.au/data-dictionaries) | January 2015-December 2019 | VIC, TAS, NSW, ACT |
|  | [Emergency Department Data Collection](https://www.cherel.org.au/data-dictionaries) | January 2015-December 2019 | VIC, TAS, NSW, ACT |
| Centre for Health Record Linkage | [Admitted Patient Data Collection](https://www.cherel.org.au/data-dictionaries) (NSW) | January 2015-June 2019 | VIC, TAS, NSW, ACT |
|  | [Emergency Department Data Collection](https://www.cherel.org.au/data-dictionaries) (NSW) | January 2015-June 2019 | VIC, TAS, NSW, ACT |
|  | [Admitted Patient Data Collection](https://www.cherel.org.au/data-dictionaries) (ACT) | January 2015-June 2019 | VIC, TAS, NSW, ACT |
|  | [Emergency Department Data Collection](https://www.cherel.org.au/data-dictionaries) (ACT) | January 2015-June 2019 | VIC, TAS, NSW, ACT |

**Recruitment methods**

**Australian IPF Registry**

The Australian Idiopathic Pulmonary Fibrosis Registry (AIPFR) was established in 2012. It is a multi-centre, prospective, observational registry of incident and prevalent IPF patients from every State and Territory in Australia. The registry is an opt-in registry and, collects serial data and clinical investigations from participants and physicians and operates in tandem with a linked biobank. Recruitment is not limited to tertiary institutions and all pulmonologists operating in Australia can refer patients with a diagnosis of IPF to the Registry. Once participants have consented to the Registry, the IPF diagnosis is re-evaluated by a multidisciplinary panel and the diagnosis is classified based on American Thoracic Society/European Respiratory Society/Japanese Respiratory Society/Latin American Thoracic Association (ATS/ERS/JRS/ALAT) guidelines. Baseline data are also collected via predesigned proformas from the participants and clinicians, and thenceforth every 6 months. The 6 monthly follow up data collected includes participant and physician proformas, medical progress, examination findings as well as results of investigations performed. All data is remotely entered into a central database managed by the Lung Foundation of Australia.

**Study participants**

Inclusion criteria for this study were: a current diagnosis of IPF, enrolment in the Australian IPF Registry, aged 18 years and older and ability to provide informed consent. All participants were invited to consent and once consented were provided with a questionnaire for completion which collected socio-demographic information, information on comorbidities, and treatment. Participants also consented to access to their administrative data arising from the interface with healthcare facilities.

**Supplement S2: Results**

Table S2 Comparison of participants and non-responders to the survey

|  | **Non-responders** | **Participants** | **p value** |
| --- | --- | --- | --- |
|  | **(n=126)** | **(n=162)** |  |
| **Age (years)** |  |  | 0.20 |
| Mean (SD) | 75.2 (9.1) | 73.9 (7.5) |  |
| **Age group, years, n (%)** |  |  | **0.00** |
| <65 | 18 (14.3) | 18 (11.1) |  |
| 65 75 | 38 (30.2) | 83 (51.2) |  |
| 75 85 | 57 (45.2) | 48 (29.6) |  |
| 85+ | 13 (10.3) | 13 (8.0) |  |
| **Sex, n (%)** |  |  | 0.91 |
| Male | 81 (64.3) | 102 (63.0) |  |
| Female | 45 (35.7) | 60 (37.0) |  |
| **Ethnicity, n (%)** |  |  | 0.34 |
| Caucasian | 111 (88.1) | 145 (89.5) |  |
| Other | 3 (2.4) | 9 (5.6) |  |
| **Comorbidities, n (%)** |  |  | **0.04** |
| No | 13 (10.3) | 33 (20.4) |  |
| Yes | 113 (89.7) | 129(79.6) |  |

n, number; %, percentage; SD, standard deviation; Bolded results represent statistically significant results (p<0.05).

Table S3 Comorbidity profile of participants

| **Comorbidity type/group** | **n (%)** |
| --- | --- |
| Musculoskeletal | 65 (40) |
| Circulatory | 47 (29) |
| Digestive | 31 (19) |
| Diabetes | 28 (17) |
| Asthma | 22 (14) |
| Other comorbidities | 17 (10) |
| Cancer | 12 (7) |
| Hypertension | 11 (7) |
| Stroke | 9 (6) |
| Kidney disease | 7 (4) |
| Liver disease | 7 (4) |
| Mental health | 4 (2) |
| Other respiratory system diseases | 3 (2) |
| Autoimmune diseases | 3 (2) |

n, number; %, percentage;

Table S4 Predicted total per person costs for a 12-month period corresponding to males aged 65-75 years with IPF in Australia

|  | **Mean costs per person, $ (95% CI)** | | | | |  |
| --- | --- | --- | --- | --- | --- | --- |
|  | **Comorbidities** | | **Medications** | | |  |
|  | **No** | **Yes** | | **Antifibrotic medication (Yes)** | **Antifibrotic medication (No)** | |
| **GAP Stage** |  |  | |  |  | |
| GAP Stage 1 | 22,815 (15,367-33,871) | 32,349 (22,483-46,543) | | 37,223 (26,197-52,892) | 15,250 (10,252-22,685) | |
| GAP Stage 2 | 26,704 (18,571-38,397) | 37,863 (28,572-50,176) | | 45,646 (34,464-60,458) | 18,701 (13,354-26,188) | |
| GAP Stage 3 | 32,252 (18,377-56,602) | 45,730 (26,118-80,068) | | 44,721 (25,824-77,443) | 18,321 (10,355-32,418) | |
| **FVC% Classification** |  |  | |  |  | |
| FVC>75 | 25,124 (17,996-35,075) | 35,328 (26,839-46,502) | | 39,852 (30,709-51,717) | 15,527 (11,259-21,412) | |
| FVC 50-75 | 25,947 (16,925-39,778) | 36,485 (25,574-52,053) | | 51,714 (35,934-74,424) | 20,148 (13,787-29,445) | |
| FVC<50 | 30,784 (13,032-72,719) | 43,287 (18,170-103,127) | | 39,929 (17,073-93,384) | 15,557 (6,547-36,965) | |
| **CPI Classification** |  |  | |  |  | |
| CPI≤ 40 | 21,852 (14,976-31,884) | 29,441 (20,818-41,637) | | 32,401 (23,277-45,100) | 12,973 (8,986-18,729) | |
| CPI>40 | 29,499 (20,744-41,950) | 39,744 (30,150-52,392) | | 48,323 (36,999-63,113) | 19,348 (13,921-26,890) | |

CI, confidence intervals; FVC, forced vital capacity percent predicted; GAP, Gender, Age, Physiology; CPI, Composite Physiological Index.

Table S5 Comparison of study estimated direct costs with IPF costs in other countries and costs for other common diseases in older people

| **Study** | **Cost year** | **Country** | **Currency** | | ***Annual cost per person (CPP)**  **$ (2021)** | | ****National per capita health expenditure (NHPCE)**  **$ (2021)** | **CPP/NHPCE** | |
| --- | --- | --- | --- | --- | --- | --- | --- | --- | --- |
| **Study estimate** | **2021** | **Australia** | | **$** | **31,655** | | **7,927** | | **4** |
| **Comparison with other countries** | | | | | | | | | |
| Coral et al (2020) [38] | 2015 | USA | US dollars | | | 204,079-244,277 | 14,764 | 13.8-16.5 | |
| Kalluri et al (2020) [39] | 2018 | Canada | Canadian dollars | | | 33,791-46,727 | 7,132 | 4.7-6.6 | |
| Marijic et al (2021) [40] | NR | Germany | Euros | | | 56,236-57,680 | 8,045 | 7.0-7.2 | |
| [National](https://clinicaltrials.gov/ct2/show/results/NCT03386994) Institute for Health (2020) [42] | NR | Spain | Euros | | | 33,578-61,825 | 4,023 | 8.3-15.4 | |
| Naoum et al (2017) [41] | 2016 | Greece | Euros | | | 39,362 | 2,294 | 17.2 | |
| Spagnolo et al (2020 [43] | 2016 | France | Euros | | | 48,792-50,365 | 6,579 | 7.4-7.7 | |
| Strens et al (2020) [44] | 2020 | Belgium | Euros | | | 40,267 | 7,946 | 5.1 | |
| **Comparison with other diseases** | | | | | | | | | |
| Asthma^‡^ [47] | 2015 | Australia | $ | | | 12,797 | 7,552 | 1.7 | |
| Cardiovascular disease^§^ [49] | 2015 | Australia | $ | | | 7,942 | 7,552 | 1.1 | |
| COPD [47] | 2008 | Australia | $ | | | 9,531 | 7,564 | 1.3 | |
| Lung Cancer^†^ [48] | 2013 | Australia | $ | | | 59,216 | 9,448 | 6.3 | |
| Osteoarthritis [46] | 2015 | Australia | $ | | | 1,057 | 7,552 | 0.1 | |
| Type 2 Diabetes^§^ [47] | 2015 | Australia | $ | | | 1,131 | 7,552 | 1.1 | |

All studies are cover periods after antifibrotic treatment was introduced.

Where a cost range is given, this represents costs from mild disease to severe disease.

*Where the cost year was not given, we assumed costs were inflated to the year of publication of the study.

**National per capita health expenditure was aligned to the cost year.

^‡^Direct and indirect costs

^§^ Direct medical costs

^†^Medicare, Pharmaceutical benefits scheme and hospital costs. Costs are excess costs of lung cancer compared to controls without cancer
